# Supplementary material for: Fast and interpretable quantification of biological shape heterogeneity via stratified Wasserstein kernel
Source: PLoS Comput Biol. 2026 May 7;22(5):e1014254. doi: 10.1371/journal.pcbi.1014254 (PMC13167030; doi:10.1371/journal.pcbi.1014254)
Supplement: S1 Text — (PDF) [file pcbi.1014254.s001.pdf]

Supporting Information S1 text

Fast and interpretable quantification of biological shape heterogeneity via stratified Wasserstein kernel

1 Description and properties of stratified Wasserstein distance

Definition 1.1 (Local distance distribution) *For each shape $\mathcal{S}$ and point $x \in S$, the local distance distribution is denoted as:*

$$\nu_x^{\mathcal{S}} := (d_{\mathcal{S}}(x, \cdot))_{\#}\mu_{\mathcal{S}}.$$

It describes the distribution of all distances $d(x, y)$ from the reference point x to any $y \in \mathcal{S}$, and is supported in a bounded interval $[0, \mathrm{diam}(\mathcal{S})]$. From the theory of moment-generating functions, each 1-D distribution is fully determined from all of its moments $m^k(x)$ for $k = 1, 2, ...$, and the existence of moments is guaranteed from the compact support. Moreover, the local distances have no atoms, from the fact that all shapes are continuous.

First, we assume that an oracle ranking function r that labels all points is known a priori. This is reasonable, for instance, given that the local distance distributions $\nu_x^{\mathcal{S}}$ are uniquely determined by their moment sequences $\{m_i^{\mathcal{S}}(x)\}_{i\geq 1}$, so a functional that achieves lexicographical ordering according to moments would satisfy the criteria:

Hypothesis (H1) (Ranking functional) *There exists a measurable functional $\psi : \mathcal{P}([0, diam(\mathcal{S})]) \to \mathbb{R}$ such that $r^{\mathcal{S}}(x) = \psi(\nu_x^{\mathcal{S}})$ and:*

- *(i) (*Continuity*) ψ is continuous at $\nu_x^{\mathcal{S}}$ for $\mu_{\mathcal{S}}$-a.e. x with respect to weak (equivalently W_1) convergence on $\mathcal{P}([0, diam(\mathcal{S})])$;*
- *(ii) (*A.e. injectivity*) $r^{\mathcal{S}}$ is injective $\mu_{\mathcal{S}}$-a.e.;*
- *(iii) (*Non-atomic pushforward*) $r^{\mathcal{S}}\#\mu_{\mathcal{S}}$ has no atoms, so $U = F(r)$ is uniformly distributed on $[0, 1]$;*
- *(iv) (*Empirical stability*) For the empirical law $\hat{\nu}_x$ built from distances, $\psi(\hat{\nu}_x) \to \psi(\nu_x)$ a.s. for $\mu_{\mathcal{S}}$-a.e. x (hence $\hat{r}_{n\#}\mu_n \Rightarrow r_{\#}\mu$).*

Such an oracle ranking function does not exist in practice, yet it is always possible to construct one when finite points are available for each shape. Below we propose an empirical surrogate which can be constructed based on finite points and moments of their local distance distributions:

Theorem 1 (Ranking by moments) *For any shape $\mathcal{S}$, the local distance distributions $\nu_x^{\mathcal{S}}$ are uniquely determined by their moment sequences $\{m_i^{\mathcal{S}}(x)\}_{i\geq 1}$. Moreover, for any finite set of points $\{x_a\}$ and for almost every $\epsilon > 0$, the polynomial*

$$r^{\mathcal{S}}(x, \epsilon) = \sum_{i=1}^{M} m_i^{\mathcal{S}}(x)\, \epsilon^{i-1}$$

is injective on $\{x_a\}$ whenever the corresponding distributions $\nu_{x_a}^{\mathcal{S}}$ are distinct.

Proof. Moment determinacy on compact intervals ensures that different $\nu_x^{\mathcal{S}}$ differ in some finite prefix of moments. For such a pair (x, y) the difference $r^{\mathcal{S}}(x, \epsilon) - r^{\mathcal{S}}(y, \epsilon)$ is a nonzero polynomial with finitely many roots. Excluding these exceptional ϵ's, the map $r^{\mathcal{S}}(\cdot, \epsilon)$ is injective on the finite sample. ∎

Remark 1.1: When ϵ is small, this is similar to hierarchical sorting given the moments according to its order. First, we sort all points in $\mathcal{S}$ by $m_1(x)$, mean distance to all other points. In case of tie, say, there exists a set of points that have equal $m_1(x)$, the points are further sorted according to $m_2(x)$. Recursively, this will determine an order of all points on the shape, unless there are identical local distance distributions (and it is not necessary to determine the order between them any more).

Remark 1.2: The remark assumes no asymmetry. In real data with noise, to distinguish finitely many shapes, each with finitely many points, it often suffices to take $M = 1$ and $\epsilon = 0$, so the ranking function only looks at the average distance to all other points.

Using the ranking functional, we can stratify the shape and construct an embedding from it into the Euclidean space, described below.

Definition 1.2 (Stratified Wasserstein distance) *Let* $U^{\mathcal{S}}(x) = F(r^{\mathcal{S}}(x, \epsilon))$ *be the normalized rank, uniformly distributed over* $[0, 1]$ *within each shape. We first disintegrate each shape measure along* $U^{\mathcal{S}}$*:*

$$\mu_{\mathcal{S}} = \int_0^1 \mu_{\mathcal{S}}^u du, \quad \mu_{\mathcal{S}}^u = \mu_{\mathcal{S}}(\,\cdot\,|U = u), \quad u \in [0, 1].$$

Then we embed each shape using:

$$\Phi_{\mathcal{S}}(u, q) := Q\left(\nu^{\mathcal{S}}_{\mu^u_{\mathcal{S}}}, q\right) = \int Q(\nu_x^{\mathcal{S}}, q)\mu_{\mathcal{S}}^u(x)\; dx = \mathbb{E}_{\mu_{\mathcal{S}}^u}[Q(\nu_x^{\mathcal{S}}, q)], \quad u \in [0, 1], \quad q \in [0, 1],$$

where $Q(\cdot, q)$ *is the* q*-th quantile of the local distance distribution. For any shape* $\mathcal{S}$*, the embeddings* $\Phi_{\mathcal{S}} \in L^2([0, 1]^2)$ *from the boundedness of* $\nu_x^{\mathcal{S}}$*. The stratified distance is defined as the Euclidean distance in the embedding space:*

$$D^2(\mathcal{S}_1, \mathcal{S}_2) = \int_0^1 \int_0^1 |\Phi_{\mathcal{S}_1}(u, q) - \Phi_{\mathcal{S}_2}(u, q)|_2^2 \; du \; dq.$$

Theorem 2 (Injectivity) *The stratified distance* $D^2(\mathcal{S}_1, \mathcal{S}_2)$ *equals zero if and only if* $\mathcal{S}_1$ *and* $\mathcal{S}_2$ *are identical up to isometry.*

Proof. ($\Rightarrow$) If $\mathcal{S}_1$ and $\mathcal{S}_2$ are isometric, their local distance distributions coincide, hence the embeddings Φ coincide pointwise, so $D^2(\mathcal{S}_1, \mathcal{S}_2) = 0$.

($\Leftarrow$) Suppose $D^2(\mathcal{S}_1, \mathcal{S}_2) = 0$. Define the rank–preserving coupling

$$\gamma^{\text{stratified}} = \int_0^1 \pi_u \, du, \qquad \pi_u \in \Pi(\mu_1^u, \mu_2^u).$$

Following Hypothesis (H1)(ii)–(iii), the disintegrations μ_1^u, μ_2^u are Dirac almost everywhere, so each π_u reduces to a dirac mass matching the unique points of rank u in $\mathcal{S}_1$ and $\mathcal{S}_2$.

For this plan we have

$$0 = D^2(\mathcal{S}_1, \mathcal{S}_2) = \iint W_2^2(\nu_x^{\mathcal{S}_1}, \nu_y^{\mathcal{S}_2})\, \gamma^{\text{stratified}}(x, y)\, dx\, dy$$

It is an upper bound of the second lower bound of Gromov–Wasserstein, minimized over all possible couplings:

$$\text{SLB}(\mathcal{S}_1, \mathcal{S}_2) = \inf_{\gamma} \iint W_2^2(\nu_x^{\mathcal{S}_1}, \nu_y^{\mathcal{S}_2})\gamma(x, y)\; dx\; dy \le \iint W_2^2(\nu_x^{\mathcal{S}_1}, \nu_y^{\mathcal{S}_2})\, \gamma^{\text{stratified}}(x, y)\, dx\, dy.$$

Thus the second lower bound of Gromov–Wasserstein distance is 0, and by its injectivity property [1], $\mathcal{S}_1$ and $\mathcal{S}_2$ must be isometric. ∎

References

[1] F. Mémoli and T. Needham. Distance distributions and inverse problems for metric measure spaces. *Studies in Applied Mathematics*, 149(4):943–1001, Aug. 2022.
