# Supplementary material for: Fast and interpretable quantification of biological shape heterogeneity via stratified Wasserstein kernel
Source: PLoS Comput Biol. 2026 May 7;22(5):e1014254. doi: 10.1371/journal.pcbi.1014254 (PMC13167030; doi:10.1371/journal.pcbi.1014254)
Supplement: S2 Text — (PDF) [file pcbi.1014254.s002.pdf]

Supporting Information S2 text

Fast and interpretable quantification of biological shape heterogeneity via stratified Wasserstein kernel

Properties on stratified Wasserstein kernels

A Gaussian kernel defined by

$$k_\sigma(\mathcal{S}_1, \mathcal{S}_2) := \exp\big(-\tfrac{1}{2\sigma^2} \iint |\Phi_{\mathcal{S}_1}(u,q) - \Phi_{\mathcal{S}_2}(u,q)|_2^2 \, du \, dq \,\big)$$

is positive semidefinite for all $\sigma > 0$ by the standard result that the Gaussian of a Hilbertian metric is PSD. Since Φ maps into a Euclidean space, $\|\cdot\|_2$ is Hilbertian, hence k_σ is PSD.

If the embedding Φ is injective (cf. previous section), then k_σ is characteristic, i.e., it metrizes weak convergence of probability measures over the shape space when shapes are sampled i.i.d. from a distribution. Similarly, this extends to other kernels that are characteristic in Euclidean space, including Laplacian kernel and Matern kernels [1].

1 Sample-based approximation is consistent

First, we assume that an oracle ranking function r that labels all points is known a priori. This is reasonable, for instance, given that the local distance distributions $\nu_x^{\mathcal{S}}$ are uniquely determined by their moment sequences $\{m_i^{\mathcal{S}}(x)\}_{i\geq 1}$, so a functional that achieves lexicographical ordering according to moments would satisfy the criteria:

Hypothesis (H1) (Ranking functional) *There exists a measurable functional* $\psi : \mathcal{P}([0, \mathit{diam}(\mathcal{S})]) \to \mathbb{R}$ *such that* $r^{\mathcal{S}}(x) = \psi(\nu_x^{\mathcal{S}})$ *and:*

*(i) (*Continuity*)* ψ *is continuous at* $\nu_x^{\mathcal{S}}$ *for* $\mu_{\mathcal{S}}$*-a.e.* x *with respect to weak (equivalently* W_1*) convergence on* $\mathcal{P}([0, \mathit{diam}(\mathcal{S})])$*;*

*(ii) (*A.e. injectivity*)* $r^{\mathcal{S}}$ *is injective* $\mu_{\mathcal{S}}$*-a.e.;*

*(iii) (*Non-atomic pushforward*)* $r^{\mathcal{S}}\#\mu_{\mathcal{S}}$ *has no atoms, so* $U = F(r)$ *is uniformly distributed on* $[0,1]$*;*

*(iv) (*Empirical stability*) For the empirical law* $\hat{\nu}_x$ *built from distances,* $\psi(\hat{\nu}_x) \to \psi(\nu_x)$ *a.s. for* $\mu_{\mathcal{S}}$*-a.e.* x *(hence* $\hat{r}_{n\#}\mu_n \Rightarrow r_{\#}\mu$*).*

Hypothesis (H2) (Bin growth) *With* n *samples per shape, each divided into* K *equal-mass bins according to* r*,* $\min_k |B_{i,k}| \to \infty$ *for* $i = 1, 2$ *so each bin gets a sufficiently large number of points.*

Theorem 1 (Consistency of the stratified distance) *Assume the existence and regularity of an oracle ranking functional (H1) and Bin Growth (H2). Let* $D^2(\mathcal{S}_1, \mathcal{S}_2) = \int_0^1\int_0^1 |\Phi_{\mathcal{S}_1}(u,q) - \Phi_{\mathcal{S}_2}(u,q)|^2 \, dq \, du$ *denote the population distance,* $D^2_{K,L} = \frac{1}{KL}\sum_{k,l} \|\phi_{\mathcal{S}_1}(u_k, q_l) - \phi_{\mathcal{S}_2}(u_k, q_l)\|_2^2$*, and let* $\widehat{D}^2_{n,K,L}$ *be its empirical approximation with* K *equal-mass* u*-bins and an* L*-point* q*-grid computed when each shape has only* n *points observed.*

(a) ***Fixed discretization.*** *For any fixed* K, L, $\widehat{D}^2_{n,K,L} \xrightarrow{a.s.} D^2_{K,L}$ *as* $n \to \infty$, *where* $D^2_{K,L}$ *is the corresponding discretized population functional.*

(b) ***Refining discretization.*** *As grid size* $1/L = \max_\ell (q_{\ell+1} - q_\ell) \to 0$ *(refining* q*) and* $K \to \infty$ *(refining* u*),* $D^2_{K,L} \to D^2(\mathcal{S}_1, \mathcal{S}_2)$.

(c) ***Joint regime.*** *If* $K = K(n) \to \infty$ *and* $L = L(n) \to \infty$ *with* $n/K(n) = \min_k |B_{i,k}| \to \infty$, *then*

$$\widehat{D}^2_{n,K(n),L(n)} \xrightarrow{\mathbb{P}} D^2(\mathcal{S}_1, \mathcal{S}_2).$$

Remark 1.1 *Part (a) ensures that with fixed small* K, L *the estimator is already consistent for its discretized target; parts (b)–(c) show that refining* K, L *recovers the continuous population distance.*

Proof of consistency Under Hypothesis (H1) and (H2), we aim to prove (a)-(c) as stated above.

Step 1: Fixed K, L. Let $U = F(r)$ be the normalized rank. Equal-mass empirical bins $B_{i,k}$ are order-statistic blocks in U; by Rank/Bins and Bin Growth, $n_{i,k} \to \infty$ and the empirical conditional law in $B_{i,k}$ converges to the population conditional $\mu^{I_k}_{\mathcal{S}_i}$. For each fixed q_ℓ, the atomless, bounded-support assumption implies $\widehat{Q}(\nu_x, q_\ell) \to Q(\nu_x, q_\ell)$ for every x (Glivenko–Cantelli Theorem). By the strong law within each bin,

$$\widehat{\Phi}_{\mathcal{S}_i,n}(I_k, q_\ell) = \frac{1}{n_{i,k}} \sum_{x \in B_{i,k}} \widehat{Q}(\nu_x, q_\ell) \xrightarrow{\text{a.s.}} \int Q(\nu_x, q_\ell)\, d\mu^{I_k}_{\mathcal{S}_i}(x) = \overline{\Phi}_{\mathcal{S}_i}(I_k, q_\ell).$$

Since there are finitely many (k, ℓ), we obtain $\widehat{D}^2_{n,K,L} \to D^2_{K,L}$.

Step 2: Refining q. For each fixed bin I_k, the map $q \mapsto \overline{\Phi}_{\mathcal{S}_i}(I_k, q)$ is bounded and monotone (average of quantile functions). Hence, as the mesh of the q-grid goes to zero, the Riemann sums converge to the integral:

$$D^2_{K,L} \;\longrightarrow\; D^2_K := \frac{1}{K} \sum_{k=1}^{K} \int_0^1 \left| \overline{\Phi}_{\mathcal{S}_1}(I_k, q) - \overline{\Phi}_{\mathcal{S}_2}(I_k, q) \right|^2 dq.$$

Step 3: Refining u. Let $G(u, q) := \Phi_{\mathcal{S}_1}(u, q) - \Phi_{\mathcal{S}_2}(u, q) \in L^2([0,1]^2)$. For each q, the bin-average $\overline{G}_K(I_k, q) := \int_{I_k} G(u, q)\, K\, du$ is the conditional expectation $\mathbb{E}[G(U, q) \mid \sigma(\{I_k\})]$. By the martingale convergence theorem for refining partitions, $\overline{G}_K(\cdot, q) \to G(\cdot, q)$ in $L^2([0,1])$ as $K \to \infty$. Integrating in q and using dominated convergence yields $D^2_K \to D^2(\mathcal{S}_1, \mathcal{S}_2)$.

Step 4: Joint regime. For $K = K(n)$ and $L = L(n)$, decompose

$$\left| \widehat{D}^2_{n,K,L} - D^2 \right| \le \underbrace{\left| \widehat{D}^2_{n,K,L} - D^2_{K,L} \right|}_{\text{estimation}} + \underbrace{\left| D^2_{K,L} - D^2_K \right|}_{q\text{-discretization}} + \underbrace{\left| D^2_K - D^2 \right|}_{u\text{-discretization}}.$$

The estimation term $\to 0$ by Step 1 applied with growing bin sizes (Hypothesis (H2)) and law of large numbers. The q- and u-discretization terms vanish by Steps 2 and 3 when the $L \to \infty$ and $K \to \infty$. Therefore $\widehat{D}^2_{n,K(n),L(n)} \to D^2(\mathcal{S}_1, \mathcal{S}_2)$. ∎

References

[1] D. Sejdinovic, B. Sriperumbudur, A. Gretton, and K. Fukumizu. Equivalence of distance-based and rkhs-based statistics in hypothesis testing. *The Annals of Statistics*, 41(5), Oct. 2013.
