# Supplementary material for: Fast and interpretable quantification of biological shape heterogeneity via stratified Wasserstein kernel
Source: PLoS Comput Biol. 2026 May 7;22(5):e1014254. doi: 10.1371/journal.pcbi.1014254 (PMC13167030; doi:10.1371/journal.pcbi.1014254)
Supplement: S3 Text — (PDF) [file pcbi.1014254.s003.pdf]

Supporting Information S3 text

Fast and interpretable quantification of biological shape heterogeneity via stratified Wasserstein kernel

Examples

This section has simple, 2D examples to help clarify how stratified distance works.

1 Difference between Wasserstein on global vs local distributions

Here we use a simple example taken from [1, 2] to illustrate the difference between local and global distance profiles:

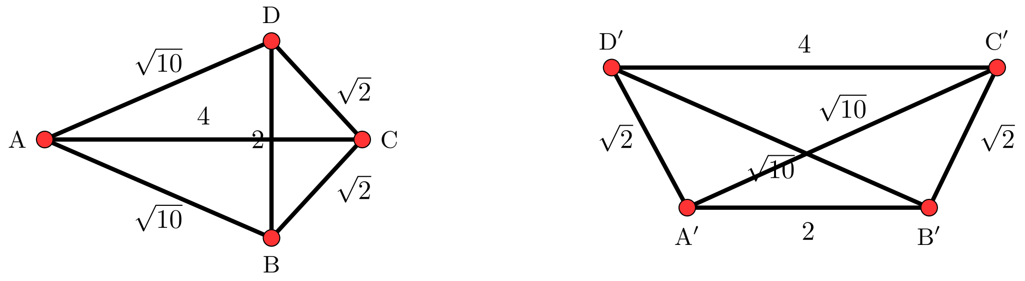

Figure A: *Illustration on global and local distance metrics*

The two shapes have distance matrices as below:

$$C^1 = \begin{bmatrix} 0 & \sqrt{10} & 4 & \sqrt{10} \\ \sqrt{10} & 0 & \sqrt{2} & 2 \\ 4 & \sqrt{2} & 0 & \sqrt{2} \\ \sqrt{10} & 2 & \sqrt{2} & 0 \end{bmatrix}, \quad C^2 = \begin{bmatrix} 0 & 2 & \sqrt{10} & \sqrt{2} \\ 2 & 0 & \sqrt{2} & \sqrt{10} \\ \sqrt{10} & \sqrt{2} & 0 & 4 \\ \sqrt{2} & \sqrt{10} & 4 & 0 \end{bmatrix}.$$

Global distances: thinking about global distances, both are uniformly distributed over $[\sqrt{2}, \sqrt{2}, 2, \sqrt{10}, \sqrt{10}, 4]$, so the Wasserstein distance between global distances vanishes between these two shapes, indicating that the global Wasserstein distance cannot discriminate all shape spaces up to isometry.

Local distances: As a first step, the points should be ranked to create a correspondence. Sorted by average distance to all other points (descending order), the first shape results in A, C, B, D (with B and D interchangeable). The second shape is ordered by C', D', A', B', with C' and D' interchangeable and A' and B' interchangeable. As the local distances between A and C' are already non-identical, the distance is nonzero.

2 An example when sorting by mean can fail

In Figure B, we show a five-point T-shape. For $a \approx 2.2666$ the mean distance from the center C to the other points equals that from the right-arm point A, even though their local distance distributions differ. Including

the second order information (variance) can help to tell that A is more likely to be on the ‘extreme’ side than C from its high variance.

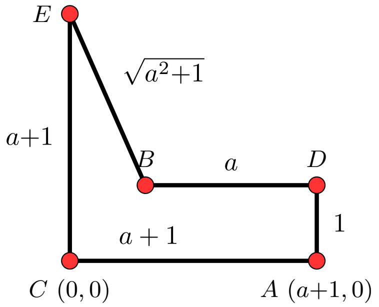

Figure B: *An example illustrating the effect of using information beyond the mean of local distance distributions.*

3 A comparison between all distances on random polygons

The second lower bound as well as the global Wasserstein distance over pairwise distances are both known to be a lower bound of Gromov–Wasserstein distance. The proposed stratified Wasserstein distance is an upper bound of the second lower bound, per the proof of injectivity. In this example, we perform a systematic comparison between these choices of distances on 200 random polygons with 6 sides, where the vertices are randomly sampled within the unit square. For the 200×200 pairs, we compute the distances between each pair of them, and visualize how the distances compare against each other in Figure C.

As expected, the global Wasserstein distance and second lower bounds (SLB) do not exceed Gromov–Wasserstein distance, while the second lower bound offers greater power of discriminating the shape differences and have typically higher values compared to the global Wasserstein. This is consistent with the remark made by [3] that the second lower bound provides an upper bound of the global distance.

The stratified Wasserstein provides an upper bound on the lower bound of Gromov–Wasserstein distance, so in principle, there is no definite conclusion on the relationship between them. We remark that the stratified Wasserstein mostly serves as a lower bound of Gromov–Wasserstein, with very few rare cases of exceeding Gromov–Wasserstein, as shown in the middle panel of Figure C. This agrees with the test power reported in the 2D cell contour example in Figure 2, panel (f) that stratified Wasserstein distance has slightly weaker test power.

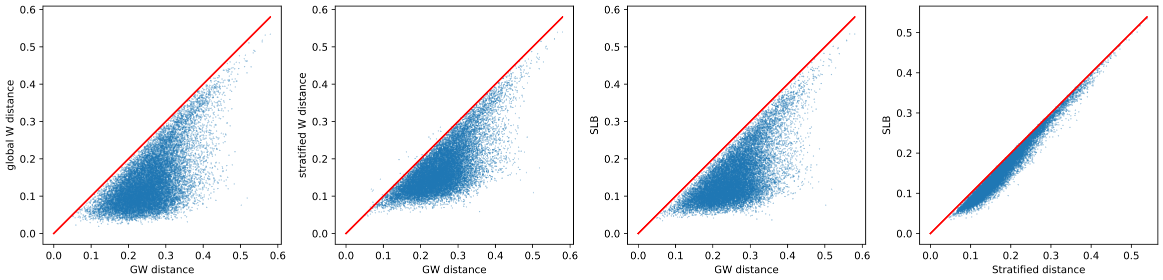

Figure C: *Comparison of distances across* 200×200 *pairs of random 6-sided polygons.*

4 Effect of imperfect matching by stratified functional

Next, we manufacture an example where the incorrect matching for points can result in large deviations from the Gromov–Wasserstein distance and inflate the distance between shapes even if they are similar. Consider T-shapes with three equal arms, due to the symmetry, ranking by the mean distance will not yield the globally optimal coupling, as shown in the top right panel of Figure D. As a result, the stratified distance is significantly greater than the second lower bound, and can still have relatively higher values even if the Gromov–Wasserstein distance is low. This suggests that stratified Wasserstein can over-penalize on the discrepancy if the functional (such as mean distance) does not yield appropriate correspondence between shapes, though such data with symmetry rarely seen in large-scale biological applications.

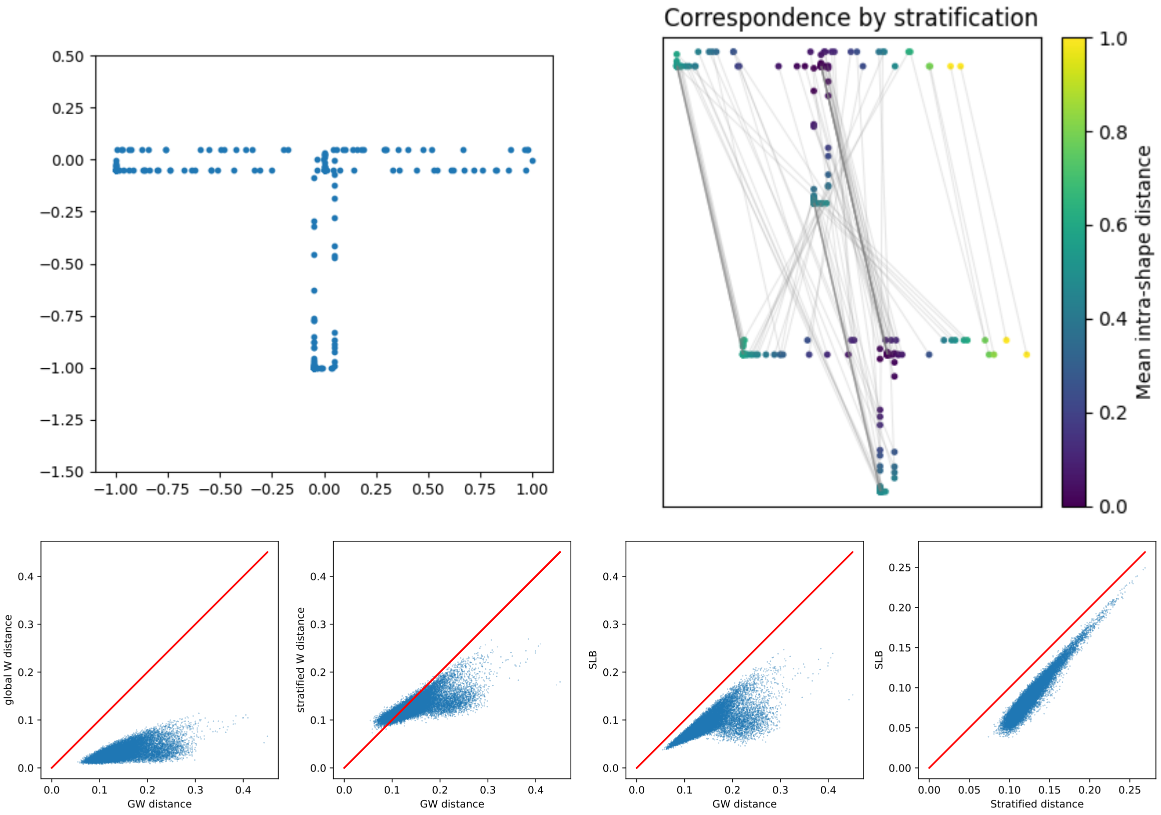

Figure D: *Top: T-shapes and example on false coupling by ranking the mean distance. Bottom: comparison across distances between* 200×200 *pairs of T-shapes with 100 randomly sampled points each.*

References

[1] M. Boutin and G. Kemper. On reconstructing n-point configurations from the distribution of distances or areas. *Advances in Applied Mathematics*, 32(4):709–735, May 2004.

[2] F. Mémoli. Gromov–wasserstein distances and the metric approach to object matching. *Foundations of Computational Mathematics*, 11(4):417–487, Apr. 2011.

[3] F. Mémoli and T. Needham. Distance distributions and inverse problems for metric measure spaces. *Studies in Applied Mathematics*, 149(4):943–1001, Aug. 2022.
